# Pneumococcal nasopharyngeal carriage and antimicrobial susceptibility profile in children under five in southern Ethiopia

**DOI:** 10.12688/f1000research.27583.1

**Published:** 2020-12-16

**Authors:** Siraj Hussen, Solomon Asnake, Demelash Wachamo, Birkneh Tilahun Tadesse

**Affiliations:** 1 School of medical laboratory, Hawassa University, Hawassa, Sidama National Regional State, 1560, Ethiopia; 2 Department of Public Health, Hawassa College of Health Sciences, Hawassa, Sidama National Regional State, 84, Ethiopia; 3 School of Medicine, Hawassa University, Hawassa, Sidama National Regional State, 1560, Ethiopia

**Keywords:** Nasopharyngeal carriage, Streptococcus pneumonia, antimicrobial susceptibility, under-five children, Ethiopia

## Abstract

**Background**: *Streptococcus pneumonia* causes high morbidity and mortality, particularly in children under five. Nasopharyngeal (NP) carriage predisposes individuals to pneumococcal infection and horizontal spread within the community. Overuse of antibiotics has been linked to increased risk of antimicrobial resistance to *S. pneumonia*. We investigated NP carriage rate and resistance to commonly prescribed antibiotics in under-five children visiting a public referral center in southern Ethiopia.

**Methods**: In total, 413 under 5 children who visited the outpatient department for a health check-up, immunization or acute mild illnesses underwent NP sampling. Parent/caregiver surveys were administered at the clinic. Sterile plastic applicator rayon tipped swabs were used for NP sampling. Antimicrobial susceptibility testing was performed using modified the disk diffusion method.

**Results**: *S. pneumonia* NP carriage was observed in 39% [95% confidence interval (CI): 34.4–43.8]. Living with one or more sibling (AOR (adjusted odds ratio) 1.95: 95% CI: 1.01, 3.76), age group of 3-23 months (AOR 2.31: 95% CI: 1.07, 4.98), co-sleeping with family (AOR 2.09, 95% CI: 1.16, 3.79), attendance at kindergarten/day-care (AOR 1.84: 95% CI: 1.09, 3.11) and malnutrition independently increased *S. pneumonia* carriage at the individual level. *S. pneumonia* was highly resistant to Oxacillin (38.5%), Tetracycline (37.3%), and Trimethoprim-sulfamethoxazole (34.2%). Multi-drug resistance was observed in 42.2% of isolates.

**Conclusions**: A high streptococcal NP carriage rate was observed in under-five children. The high level of resistance to commonly used antibiotics calls for enhancing national surveillance of resistance patterns and enforce antibiotic stewardship efforts.

## Abbreviations:

EDHS = Central Statistical Agency of Ethiopia, PCV = pneumococcal conjugate vaccine, NP = nasopharyngeal, STGG = skim milk tritons glucose glycerol, and *S. pneumonia = Streptococcus pneumonia.*

## Introduction

*Streptococcus pneumonia* (pneumococcus) is a Gram-positive extracellular pathogen associated with high morbidity and mortality in children all over the world, particularly in developing countries like Ethiopia ^1^. *S. pneumonia* is the most important cause of bacterial pneumonia and meningitis worldwide. For instance, in 2010, it accounted for 33% of the deaths in children under 4 years old ^2^. In Africa, pneumococcal disease is estimated to cause nearly half a million deaths among children under five years annually ^3^. The World Health Organization (WHO) in 2010 estimated 541,000 global child deaths due to pneumococcal infections in under 5 years children ^3^. Ethiopia is among the countries with the highest burden of pneumonia, especially in children under five ^4^. In 2010, 312,857 cases community acquired pneumonia and 12,284 deaths caused by *S. pneumonia* were reported in children under five ^5^.

Pneumococcal disease often follows nasopharyngeal (NP) colonization with homologous strains. The mucosal epithelium of the nasopharynx is the primary site of pneumococcal colonization ^6^. *S. pneumonia* NP carriage, a necessity for the development of the disease, is considered to be an important source of horizontal spread of this pathogen within the community ^7^.

Several socio-demographic and clinical characteristics including young age, family size, low income, number of siblings, and malnutrition predicted NP pneumococcal colonization ^8^. Household and environmental factors such as overcrowding, exposure to tobacco smoke ^9^ and exposure to indoor air pollution also increased the risk of NP colonization ^8^. The transmission of *S. pneumonia* occurs through respiratory droplets or more commonly from individuals who are asymptomatic carriers ^6^. Pneumococcus susceptible individuals may become colonized upon exposure and can remain so for weeks to months. Acquisition of invasive serotypes could lead to pneumococcal disease, commonly after a 1 to 3 day incubation period ^10^.

The increasing frequency and rapid spread of antimicrobial resistant pneumococcal strains is a global health threat. Antimicrobial resistance has made the choice of antimicrobial agents for treatment of pneumococcal infections more complicated and costly ^9, 11^. Nasopharyngeal colonization by antimicrobial resistant *S. pneumonia* had been increasing in different parts of the world including Ethiopia ^12, 13^. Minimizing NP carriage rate is an important step for prevention and control of pneumococcal disease. The variable risk factors in different populations and the risk factor differences necessitate generating evidence in various settings to better understand the factors that predispose to increased risk of exposure to *S pneumoniae*. We aimed to investigate the prevalence and predictors of NP pneumococcal colonization as well as antimicrobial susceptibility pattern of isolates in a setting where there is a high prevalence of undernutrition and low socioeconomic status.

## Methods

### Ethics approval and consent to participate

Ethical approval was obtained from the Institutional Review Board (IRB) of College of Medicine and Health Sciences, Hawassa University (IRB reference number: IRB/006/11). The purpose and importance of the study was explained to each study participants. To ensure confidentiality of participants, data collection tools were anonymous with no participant identifiers. Participants were interviewed alone to maintain privacy. All participants were not paid for the test. Informed written consent was obtained from a parent or guardian for children to participate in the study. The study incurs no cost to the study participants and were interviewed free of charge.

### Study location and sampling technique

The study was conducted between November 2018 and March 2019 at outpatient departments (OPD) of two public Hospitals – Adare and Hawassa University comprehensive specialized Hospitals (HUCSH) in Hawassa City, which were purposively selected to represent primary healthcare and referral facilities in the region; Adare General Hospital is a primary care facility while HUCSH is the main referral hospital in southern Ethiopia. In the study district the coverage of three doses of pneumococcal conjugate vaccine (PCV) coverage was at 61% in 2019 ^14^, which showed significant improvement from the 2016 reported coverage of 53% ^15^.

Sample size (n) was calculated using single proportion formula (Equation 1) assuming a prevalence (p) of 43% based on data reported in North West Ethiopia (43.8%) ^16^ with 95% confidence interval (z=1.96) and 5% precession (d), and 10% non-response rate which resulted in a sample size of 417. A systematic random sampling method was used to select participants – every *k ^th^* child was selected from a total of OPD attendees every day. The list of all children who presented to the OPDs everyday was used as a sampling frame to decide the value of *k*. Parents or legal authorized representatives were invited to participate in the study. The informed consent process was administered to those who agreed to participate in the study.

$$Equation\;1:\;n\;=\;\frac{z^{2}*p(1-p)}{d^{2}}$$

### Inclusion and exclusion criteria

Inclusion were all under-five children who visit OPDs of the two hospitals during the study period and who consent to participate in the study.

Exclusion criteria included subjects who had an illness that made nasal swabbing difficult, and those with severe respiratory problems (for example acute attack of bronchial asthma), had anatomical abnormalities of the nose (e.g. cleft palate) and who were on antibiotics in the two weeks prior to the start of the study.

### Data collection

Structured questionnaires developed for the purpose of the study, pilot tested on 5% of the sample before implementation, were used to collect information on socio-demographics, clinical data, and associated factors. Based on the results of the pilot testing of whether questions were correctly understood by the interviewers and respondents or not, the questionnaires were revised to improve clarity. The pilot testing did not reveal significant errors in the questionnaires. The tools were first developed in English (see extended data ^17^), translated to local language (see extended data ^18^), and back translated to English by an independent translator to ensure internal validity. In addition to interviews of parents/guardians, medical records of participants were reviewed to abstract past medical history. Trained data collector healthcare professionals administered the questionnaires to the parents or LAR in a quiet room; data collectors also measured child’s weight to the nearest 0.1kg and height/length to the nearest 1cm using electronic weighing scale and length/height board. Anthropometrics were then interpreted using WHO Z scores, where a score of <-2 is considered to indicate undernutrition.

### Sample collection and processing

Nasopharyngeal specimens were collected using sterile swabs in two replicates. One NP specimen was collected per child by gentle insertion of sterile flexible plastic applicator rayon tipped swab (Copan, Brescia, Italy, catalogue number: 26061), which was done by tilting slightly backwards and immobilize child’s head while gently restraining the child’s body. Once in place, the swab was rotated and left in place for five seconds to saturate the tip before slowly removing it. After collection, the sample was immediately placed in 1ml skimmed milk tritons glucose glycerol (STGG) transport media in tubes. Any excess samples were cut off before inoculating in the transport medium in tubes, after which the caps were tightened securely. The NP specimen was processed within 8 hours of collection and in cases where delay was encountered, it was stored at -20°C. Culturing the NP swab-STGG specimens was done on tryptone soy agar base (Oxoid, Basingstoke, Hampshire, England; Catalogue number: 105459). Briefly, the NP swab-STGG specimens were mixed thoroughly by vortexing for 30 seconds, 10 µl of the sample was then used to inoculate the plates and streaked using a sterile wire loop. The streaked plates were incubated into 5% CO2 incubator at 37°C for 24 hours. Plates were fully examined for any growth and the plates displaying no growth were re-incubated before being reported as negative. Identification of positive culture results was performed based on the appearance of colonies and the hemolytic pattern – small and watery growth surrounded by a greenish zone of alpha-hemolysis on the media.

### Optochin susceptibility and a bile solubility biochemical test

We employed similar microbiological methods to those reported by Gebre *et al*. ^16^. Briefly, to isolate pneumococci, suggestive colonies were sub-cultured and tested for optochin susceptibility and bile solubility. Optochin susceptible strains with ≥14mm in diameter zone of inhibition were identified as *S. pneumonia*. Next, alpha hemolytic strains with zone of inhibition <14 mm underwent bile solubility test using 2% sodium deoxycholate or bile salt base (Oxoid, Basingstoke, Hampshire, England; Catalogue number: 89904).

Bacterial cell suspension samples were prepared from freshly streaked presumed positive colonies of *S. pneumonia* in sterile normal saline. An adjusted 1ml of suspension was divided into two equal amounts of 0.5 ml in each tube. Then 0.5 ml of normal saline was added to one tube and 0.5 ml of 2% bile salt to the other tube as a test followed by incubation in 5% CO2 incubator at 37°C for up to 2 hours. A loss of turbidity in the bile tube but not in the saline control tube was considered as a positive test.

### Antimicrobial susceptibility test

Disk diffusion (modified Kirbye-Bauer) method on Mueller Hinton agar (Oxoid, Basingstoke, Hampshire, England, Catalogue number: 105437) supplemented with 5% sheep blood was employed for AST ^19^. Standard disks of commonly used antibiotics including Tetracycline – 30µg, Trimethoprim/sulfamethoxazole – 1.25+23.75µg, Oxacillin – 1µg, Chloramphenicol – 30µg, and Erythromycin – 15µg (Oxoid, Basingstoke, Hants RG24 8PW, UK) were used antimicrobial susceptibility testing of all the isolates.

Following inoculation of the bacteria suspension on the Mueller-Hinton agar plate, which is supplemented with 5% sheep blood agar, and then air drying, the antibiotic disks were dispensed aseptically using an automatic disk dispenser. Next, the plates were incubated in a 5% CO2 incubator at 37°C for 24 hours. Finally, zone diameters of growth inhibition were measured to the nearest millimeters using a ruler and were interpreted using cut-off points for each antibiotic disk, which range from 0.5µg/mL for vancomycin to 8µg/mL for gentamicin and tetracycline, in the Clinical and Laboratory Standard Institute (CLSI) result interpretive standards ^19^. Categorically, results were interpreted as susceptible, intermediate, or resistant ^20^. *S. pneumonia* ATCC 49619 provided by the Ethiopian National Quality Assurance Directorate (Catalogue number: 0947L) was used as a positive quality control strain for all procedures.

### Data analysis

Bivariate and multivariate binary logistic regression models containing sociodemographic and clinical variables to assess independent predictors of pneumococcal NP carriage were produced. Variables with p-value <0.2 in the bivariate model were included in the multivariate model. Odds ratios and 95% confidence intervals (CI) were used to measure the association between potential risk factors and occurrence of NP carriage at the individual level. Level of significance for the multivariate models was set at p-value < 0.005. Anthropometrics were assessed following standard procedures ^21^, Z-score of <-2.0 was used as a cut off to define wasting, stunting, and underweight for weight-for-height, height/length-for-age and weight-for-age assessments respectively ^22^. PCV vaccination status was assessed through interviews with parents/guardians and vaccination card. All the statistical tests were performed using Stata version 14.0 (StataCorp, Texas, USA).

## Results

### Socio-demographic characteristics

A total of 413 children participated in the study with 99.04% response rate; 226 (54.7%) were female. Age of the children ranged from 3–59 months with mean age (standard deviation – SD) of 36.63 (18.85) months. The majority, 308 (74.6%) of the children were from an urban setting; 157 (38.0%) of the parents/guardians attended primary education; and 230 (55.7%) of the parents/guardians were housewives. More than half, 215 (52.1%), of participants were from a family who had an average monthly income of USD 30 to 60 ( Table 1 ^23^).

**Table 1.** Pneumococcal nasopharyngeal carriage rate and predictors in children under five.

Variable	NP carriage rate		COR (95% Confidence Interval)	p-value	AOR (95% Confidence Interval)	p-value
	Number (%) Total tested	Number (%) Positive				
Sex Male Female	226(54.7) 187(45.3)	95(42.0) 66(35.3)	1.33(0.89, 1.98) ref	0.162 ref	1.22(0.78, 1.93) ref	0.385 ref
Age (months) 3–23 24–41 42–59	201(48.7) 105(25.4) 107(25.9)	104(51.7) 37(35.2) 20(18.7)	4.66 (2.66, 8.16) 2.37(1.26,4.44) ref	<0.001* 0.007 ref	2.31(1.07,4.98) 1.45(0.66,3.18) ref	0.032* 0.349 ref
Place of residence Rural Urban	308(74.6) 105(25.4)	115(37.3) 46(43.8)	ref 1.31(0.84, 2.05)	ref 0.241	ref 1.10(0.65,1.85)	ref 0.732
Mother/guardian education No formal education Primary education Secondary & above College and above	113(27.4) 157(38.0) 102(24.7) 41(9.9)	50(44.2) 60(38.2) 37(36.3) 14(34.1)	1.53(0.73, 3.22) 1.19(0.58, 2.45 1.09(0.51, 2.35) ref	0 .263 0 .632 0 .810 ref		
Mother/guardian occupation Employed Merchant Housewife	67(16.2) 116(28.1) 230(55.7)	23(34.3) 43(37.1) 95(41.3)	ref 1.13(.60, 2.12) 1.35(.76, 2.38)	0.710 0.305 ref		
Average family income ≤ 35 USD 36-65 USD > 65 USD	62(15.0) 215(52.1) 136(32.9)	25 (40.3) 86(40.0) 50(36.8)	1.16(.63, 2.15) 1.15(.74, 1.79) Ref	0 .632 0.545 ref		
Number of rooms in the house Single room More than one room	127(30.8) 286(69.2)	52(40.9) 109(38.1)	1.13(0.74,1.73) ref	0.586 Ref		
Family size Fewer than five Five or more	280(67.8) 133(32.2)	99(35.4) 62(46.6)	ref 1.597(1.052.43)	ref 0.029	ref 1.31(0.80, 2.13)	Ref 0.286
Number of siblings One or more None	281(68.0) 132(32.0)	135(48.0) 26(19.7)	3.77(2.31, 6.15) ref	<0.001* ref	1.95(1.01,3.76) ref	0.047* Ref
Co-sleeping with family Yes No	322(78.0) 91(22.0)	138(42.9) 23(25.3)	2.22(1.32, 3.74) ref	0.003 ref	2.09(1.16,3.79) ref	0.031* Ref
Child attending Kinder/Day care Yes No	134(32.4) 279(67.6)	76 (56.7) 85 (30.5)	2.99(1.95, 4.58) ref	<0.001* ref	1.84(1.09,3.11) ref	0.023* ref
Weight-for-Height Wasted Normal	52(12.6) 361(87.4)	29(55.8) 132(36.6)	2.19(1.22,3.94) ref	0.009*	2.17(1.07,4.34) ref	0.031* ref
Height -for-age Stunted Normal	100(24.2) 313(75.8)	57(57.0) 104(33.2)	2.66(1.68,4.22) ref	<0.001* ref	2.68(1.58,4.55) ref	<0.001*
Weight-for-age Underweight Normal	61(14.8) 352(85.2)	36(59.0) 135(35.5)	2.62(1.50,4.56) 1	0.001*	1.70(0.87,3.33) ref	0.123
Immunization status Three doses Two doses One dose Zero dose	250(60.5) 64(15.5) 16(3.9) 83(20.1)	94(37.6) 25(39.1) 6(37.5) 36(43.4)	1.00(.35, 2.85) 1.07(0.35, 3.31) ref 1.28(.42, 3.84)	0.994 0.909 ref 0.664		

USD – United states dollar; COR – Crude Odds Ratio; AOR – adjusted odds ratio; NP – nasopharyngeal; Ref – reference

### Prevalence of pneumococcal nasopharyngeal carriage

The overall prevalence of pneumococcal NP carriage rate was 39% [95% confidence interval (CI): 34.4–43.8]. The highest prevalence of NP carriage was observed in those aged 3 – 23 months (49.8%). More boys than girls had NP colonization (42.0% in girls versus 35.3% in boys). Of the study participants who lived in urban settings, 46 (43.8%) were carrier for *S. Pneumonia* ( Table 1 ^23^).

### Predictors of nasopharyngeal carriage

In bivariate analysis, sociodemographic variables including sex, place of residence, and age of the child had statistically significant association with pneumococcal NP carriage. Similarly, family factors including larger family size, presence of other siblings, and co-sleeping with other family members were predictors of NP pneumococcal colonization. Attendance at day care centers and presence of acute and chronic malnutrition were associated with an increased probability of NP Streptococcal colonization. Interestingly, level of PCV vaccination and lack of any vaccination were not associated with probability of NP pneumococcal colonization.

Next, we constructed a multivariable regression model including variables with a p-value < 0.2 in the bivariate analysis. Being under two years of age (AOR 2.31: 95% CI: 1.07, 4.98), those living with one or more siblings (AOR 1.95: 95% CI: 1.01, 3.76), history of co-sleeping with family members (AOR 2.09, 95% CI: 1.16, 3.79) and attendance at kindergarten/day care (AOR 1.84: 95% CI: 1.09, 3.11) were found to result in an increased probability of pneumococcal NP colonization ( Table 1 ^23^). Children who were stunted, 2.17(1.07,4.34); and wasting, 2.68(1.58,4.55) had a higher probability of pneumococcal colonization.

### Antimicrobial susceptibility bacterial isolates

Antimicrobial susceptibility was determined for all 161 isolates of *S. pneumonia* to six commonly prescribed antimicrobial agents: Oxacillin, tetracycline, Erythromycin, TMP-SMX, and chloramphenicol. Among tested antimicrobial agents, higher rates of *S. pneumonia* resistance was reported in Oxacillin, 62 (38.5%); Tetracycline, 60 (37.3%) and Trimethoprim-sulfamethoxazole, 55 (34.2%). Comparatively, the lowest resistance rate was exhibited by Erythromycin, 11 (6.8%); chloramphenicol, 117(10.6%); and Vancomycin, 13(8.1). multidrug resistance to two or more antimicrobials was identified in 68 (42.2%) isolates ( Table 2 ^23^).

**Table 2.** Antimicrobial susceptibility patterns of *S. pneumoniae* isolated among children visiting pediatrics OPD in governmental Hospitals Hawassa City, Southern Ethiopia in 2019.

	Susceptibility Pattern					
Anti-microbial Agents	Resistant		Intermediate		Sensitive	
	No.	(%)	No.	(%)	No.	(%)
Oxacillin	62	(38.5)	13	(8.1)	86	(53.4)
Tetracycline	60	(37.3)	17	(10.6)	84	(52.2)
Erythromycin	11	(6.8)	16	(9.9)	134	(83.2)
Trimethoprim- sulfamethoxazole	55	(34.2)	14	(8.7)	92	(57.1)
Chloramphenicol	17	(10.6)	7	(4.3)	137	(85.1)
Vancomycin	13	(8.1)	0	(0.0)	148	(91.9)

No. – Number; OPD – Outpatient Department

## Discussions

In the current study, we showed that streptococcal colonization is a common condition. The findings highlight the importance of pneumococcus as a common cause of bloodstream infections and cause septicemia, meningitis, and pneumonia ^24^. Ethiopia introduced the PCV vaccine in the expanded program of immunization (EPI) schedule for under five children since 2011 and the coverage has since increased. However, carriage rate of *S. pneumonia* and pneumococcal invasive disease remains a public health problem. The prevalence of pneumococcal nasopharyngeal carriage among children < 5 years in Hawassa City was 39% [95% confidence interval (CI): 34.4–43.8], a finding similar to reports from Jimma (43.8%) ^16^ and Gondar (41.03 %) ^25^. However, higher prevalence (64.8%) was reported in the Wolayita Zone of southern Ethiopia ^26^, which is similar to carriage rates reported in other African countries, for example 64.8% ^27^ and 65.8% of NP colonization in Kenya ^28^. The factors affecting the variabilities in the burden of streptococcal NP colonization have yet to be explored but could include differences in socioeconomic and population characteristics ^29^.

Even though the findings in this study represent health facility data in under-five children who visited the recruitment centers for mild health conditions, routine health check-up or vaccination, a community-based NP sample collection would have enabled inference in to the general population of under-five children. Furthermore, both recruitment facilities are urban hospitals which could limit representation of rural communities. However, the study included a significant proportion of rural residents since both hospitals have both urban and rural catchments.

In this study, NP carriage of *S. pneumonia* was significantly lower among children whose aged 24–41 months old, a finding similar to other studies in Arsi zone, South East Ethiopia ^30^ and Gondar, North West Ethiopia ^25^. The decline in *S. pneumonia* colonization rate with increasing age maybe due to the progressive acquisition of mucosal immunity as a result of repeated colonization by several serotypes and the potential reduction of exposure ^31^. Supporting this argument, our study showed that co-sleeping with other family and living with one or more siblings is associated with increased odds of NP colonization. Similar findings were reported in elsewhere ^26, 30^.

The high resistance to Tetracycline (37.8%) was consistent with other studies which reported in Gondar (33.2%) and Hawassa (42.6%) ^16, 31^, but was lower than prevalence of tetracycline resistant of *S. pneumonia* isolates from Wolayta Sodo (48.9%) and Jima (53.2%) ^16, 32^. The high resistance to tetracycline maybe due to widespread inappropriate prescription, which exerts selection pressure for the presence of increased tetracycline-resistant bacterial isolates. *S. pneumonia* isolates were also resistant to Trimethoprim-sulfamethoxazole and Oxacillin, which is in agreement with other reports ^16, 31^, reflecting local and regional antimicrobial use practices. Oxacillin is often used for soft tissue infections and injuries, including for road traffic accidents.

A worrying finding in our study was the high prevalence (42.2%) of multi-drug resistant *S. pneumonia* isolates (i.e. resistant to two or more drugs), which could be linked to mobile genetic units (including plasmids, gene cassettes in integrons and transposons) ^32^, lack of effective medicines, inappropriate dispensing, medication sharing, counterfeit drugs, bacterial evolution, climate changes, lack of medical practitioner with proper training, poor-quality and unhygienic sanitary conditions ^33^.

## Conclusion and Recommendation

The prevalence of pneumococcal Nasopharyngeal Carriage in the study area was high. The proportion of drug resistance to Tetracycline, Trimethoprim-sulfamethoxazole and Oxacillin was very high. Younger age, co-sleeping with family and living with one or more sibling independently predicted the probability of pneumococcal Nasopharyngeal carriage. The results of the study will have critical input to enforce antimicrobial stewardship efforts in the study area and beyond. Furthermore, surveillance of carriage and antimicrobial resistance in different populations will help to formulate targeted interventions.

## Data availability

### Underlying data

Figshare: Last Pneum Referal2.sav siraj Dem F.sav. https://doi.org/10.6084/m9.figshare.13297724.v1 ^23^

This project contains the following underlying data:

- Last Pneum Referal2.sav siraj Dem F.sav (Deidentified nasopharyngeal colonization data)

### Extended data

Figshare: Questionnaire (English Version).docx. https://doi.org/10.6084/m9.figshare.13297781.v1 ^17^

This project contains the following extended data:

- Questionnaire (English Version).docx

Figshare: Amharic version of the questionnaire. https://doi.org/10.6084/m9.figshare.13356641.v1 ^18^

This project contains the following extended data:

- Questionnaire_Translated version (Amharic).docx

Data are available under the terms of the Creative Commons Attribution 4.0 International license (CC-BY 4.0).
