# Supplementary material for: Pneumococcal nasopharyngeal carriage and antimicrobial susceptibility profile in children under five in southern Ethiopia
Source: F1000Res. 2021 Jul 6;9:1466. Originally published 2020 Dec 16. [Version 3] doi: 10.12688/f1000research.27583.3 (PMC8278251; doi:10.12688/f1000research.27583.3)
Supplement: Peer review history

# Peer review: Reviewer response for version 3

**DOI:** 10.5256/f1000research.58336.r89045

**Published:** 2021-07-13

**Authors:** Abate Yeshidinber Weldetsadik

**Affiliations:** 1 Department of Pediatrics and Child Health, St. Paul's Hospital Millennium Medical College, Addis Ababa, Ethiopia

I approve the revision.

Is the work clearly and accurately presented and does it cite the current literature?

Yes

If applicable, is the statistical analysis and its interpretation appropriate?

Yes

Are all the source data underlying the results available to ensure full reproducibility?

Yes

Is the study design appropriate and is the work technically sound?

Partly

Are the conclusions drawn adequately supported by the results?

Partly

Are sufficient details of methods and analysis provided to allow replication by others?

Yes

Reviewer Expertise:

Paediatric, paediatric pulmonary and critical care, quality of health care

I confirm that I have read this submission and believe that I have an appropriate level of expertise to confirm that it is of an acceptable scientific standard.

# Peer review: Reviewer response for version 2

**DOI:** 10.5256/f1000research.56639.r85118

**Published:** 2021-06-30

**Authors:** Ritah F. Mutagonda

**Affiliations:** 1 Department of Clinical Pharmacy and Pharmacology, Muhimbili University of Health and Allied Sciences, Dar es Salaam, Tanzania

I have understood the response to the comments raised, and I approve the manuscript to be considered for indexing.

Is the work clearly and accurately presented and does it cite the current literature?

Partly

If applicable, is the statistical analysis and its interpretation appropriate?

Yes

Are all the source data underlying the results available to ensure full reproducibility?

Yes

Is the study design appropriate and is the work technically sound?

Partly

Are the conclusions drawn adequately supported by the results?

Yes

Are sufficient details of methods and analysis provided to allow replication by others?

Yes

Reviewer Expertise:

Infectious disease particularly malaria and HIV; pharmacokinetics and pharmacogenomics studies; pharmacodynamics research.

I confirm that I have read this submission and believe that I have an appropriate level of expertise to confirm that it is of an acceptable scientific standard.

# Peer review: Reviewer response for version 2

**DOI:** 10.5256/f1000research.56639.r85117

**Published:** 2021-05-19

**Authors:** Abate Yeshidinber Weldetsadik

**Affiliations:** 1 Department of Pediatrics and Child Health, St. Paul's Hospital Millennium Medical College, Addis Ababa, Ethiopia

I have noticed most of my concerns are addressed in the revision but it didn't mention the limitations including the antibiotics tested are not the ones mostly used for streptococcal infections (penicillin and cephalosporin).

Is the work clearly and accurately presented and does it cite the current literature?

Yes

If applicable, is the statistical analysis and its interpretation appropriate?

Yes

Are all the source data underlying the results available to ensure full reproducibility?

Yes

Is the study design appropriate and is the work technically sound?

Partly

Are the conclusions drawn adequately supported by the results?

Partly

Are sufficient details of methods and analysis provided to allow replication by others?

Yes

Reviewer Expertise:

Paediatric, paediatric pulmonary and critical care, quality of health care

I confirm that I have read this submission and believe that I have an appropriate level of expertise to confirm that it is of an acceptable scientific standard, however I have significant reservations, as outlined above.

# Peer review: Reviewer response for version 1

**DOI:** 10.5256/f1000research.30484.r79335

**Published:** 2021-03-09

**Authors:** Ritah F. Mutagonda

**Affiliations:** 1 Department of Clinical Pharmacy and Pharmacology, Muhimbili University of Health and Allied Sciences, Dar es Salaam, Tanzania

This work addresses a very pertinent issue which is the prevalence of pneumococcal nasopharyngeal carriage and antimicrobial susceptibility profile in children under five in southern Ethiopia. With the increase in antimicrobial resistance which is currently a global health concern the information obtained from antimicrobial surveillance studies give us the clue of the current trends in pathogen antimicrobial resistance in different populations which will enable the development of targeted approaches to help control antimicrobial resistance. There are recent similar studies conducted in Ethiopia that are not featured in this manuscript eg. Negash *et al.* 2019 ^1^.

 There are minor comments which need to be addressed to improve the quality of the manuscript as described below:

**Abstract:**

- The summarized methodology sub-section needs to be improved a bit by adding information with regards to the study design, the study site, study duration, sampling technique and how data was analysed so that the reader can easy grasp this information when reading the abstract.
- Be consistent with the way you present information. Example in the methodology it is written ‘under 5’ while in the conclusion it is written ‘under-five’.

**Introduction:**

- Inconsistency of how you write ****under-five’ is also observed here.
- I do understand there is pneumococcal vaccine (PCV) which is given to children. This information is presented in the results section so I think it will be very informative to describe a bit in the introduction section as one of pneumococcal preventive measures. Could also describe the coverage which might have an impact on the pneumococcal carriage rate in children.

**Methodology:**

- I suggest that the Ethics approval and consent to participate sub-section should come after the data analysis part.
- Add subsection describing the study design --- analytical cross-sectional study.
- In data analysis, describe how you were able to analyse antimicrobial susceptibility test results. State the dependent and independent variables. Please remove this sentence *“PCV vaccination status was assessed through interviews with parents/guardians and vaccination card.”* It does not fit under data analysis part.

**Results:**

- Remove this sentence *“Next, we constructed a multivariable regression model including variables with a p-value < 0.2 in the bivariate analysis.”* Under the predictors of nasopharyngeal carriage part.

Is the work clearly and accurately presented and does it cite the current literature?

Partly

If applicable, is the statistical analysis and its interpretation appropriate?

Yes

Are all the source data underlying the results available to ensure full reproducibility?

Yes

Is the study design appropriate and is the work technically sound?

Partly

Are the conclusions drawn adequately supported by the results?

Yes

Are sufficient details of methods and analysis provided to allow replication by others?

Yes

Reviewer Expertise:

Infectious disease particularly malaria and HIV; pharmacokinetics and pharmacogenomics studies; pharmacodynamics research.

I confirm that I have read this submission and believe that I have an appropriate level of expertise to confirm that it is of an acceptable scientific standard, however I have significant reservations, as outlined above.

## References

[1] : Pneumococcal Carriage, Serotype Distribution, and Risk Factors in Children With Community-Acquired Pneumonia, 5 Years After Introduction of the 10-Valent Pneumococcal Conjugate Vaccine in Ethiopia. *Open Forum Infect Dis* .2019;6(6) : 10.1093/ofid/ofz259 ofz259 10.1093/ofid/ofz259 31263735PMC6592415

# Response

**Published:** 2021-05-12

**Authors:** Birkneh Tilahun Tadesse

**Affiliations:** Hawassa University, Ethiopia

We thank the reviewer very much for taking the time to review our paper and for the important suggestions. We agree with the importance of keeping consistency using such terms as “under-five” and we have revised throughout the paper to conform to that. We also appreciate the comment on including more information regarding the introduction of PCV in Ethiopia and we have included a paragraph in the introduction to address that. We have also made the requested formatting changes while trying to conform to the Journal's formatting requirements. Please note that the final formatting was based on the requirement of the Journal. The sentence regarding the regression models was included to ensure that there is a good flow.

# Peer review: Reviewer response for version 1

**DOI:** 10.5256/f1000research.30484.r76205

**Published:** 2021-01-21

**Authors:** Abate Yeshidinber Weldetsadik

**Affiliations:** 1 Department of Pediatrics and Child Health, St. Paul's Hospital Millennium Medical College, Addis Ababa, Ethiopia

Hussein and colleagues reported a study on "Pneumococcal nasopharyngeal carriage and antimicrobial susceptibility profile in children under five in southern Ethiopia". They underwent nasopharyngal swab culture in 413 children and found out that streptococcal carriage is common and especially increased in selected group of children with risk factors. They also reported high level of bacterial resistance.

 While it is similar to previous studies from similar setting, the study adds to the current knowledge in the region. However, their culture was determined for a group of antibiotics which are not primarily used to treat strept infection including penicillin and cephalosporins. The conclusion of strept infection as a common cause of sepsis and other infection, while a possibility and a clear risk, is also over-calling of the result in this context. These two limitations should be mentioned and rephrasing would make the paper more acceptable.

 Language edit is also strongly recommended.

Is the work clearly and accurately presented and does it cite the current literature?

Yes

If applicable, is the statistical analysis and its interpretation appropriate?

Yes

Are all the source data underlying the results available to ensure full reproducibility?

Yes

Is the study design appropriate and is the work technically sound?

Partly

Are the conclusions drawn adequately supported by the results?

Partly

Are sufficient details of methods and analysis provided to allow replication by others?

Yes

Reviewer Expertise:

Paediatric, paediatric pulmonary and critical care, quality of health care

I confirm that I have read this submission and believe that I have an appropriate level of expertise to confirm that it is of an acceptable scientific standard, however I have significant reservations, as outlined above.

# Response

**Published:** 2021-05-12

**Authors:** Birkneh Tilahun Tadesse

**Affiliations:** Hawassa University, Ethiopia

We thank the reviewer for taking the time to review our paper. As suggested, copyediting was done by a native speaker. We have reworded the first sentence of the discussion to indicate the possibility of severe infections following Streptococcal colonization.
